# orfipy: a fast and flexible tool for extracting ORFs

**DOI:** 10.1093/bioinformatics/btab090

**Published:** 2021-02-08

**Authors:** Urminder Singh, Eve Syrkin Wurtele

**Affiliations:** Bioinformatics and Computational Biology Program, Iowa State University, Ames, IA 50011, USA; Center for Metabolic Biology, Iowa State University, Ames, IA 50011, USA; Department of Genetics Development and Cell Biology, Iowa State University, Ames, IA 50011, USA; Bioinformatics and Computational Biology Program, Iowa State University, Ames, IA 50011, USA; Center for Metabolic Biology, Iowa State University, Ames, IA 50011, USA; Department of Genetics Development and Cell Biology, Iowa State University, Ames, IA 50011, USA

## Abstract

**Summary:**

Searching for open reading frames is a routine task and a critical step prior to annotating protein coding regions in newly sequenced genomes or *de novo* transcriptome assemblies. With the tremendous increase in genomic and transcriptomic data, faster tools are needed to handle large input datasets. These tools should be versatile enough to fine-tune search criteria and allow efficient downstream analysis. Here we present a new python based tool, orfipy, which allows the user to flexibly search for open reading frames in genomic and transcriptomic sequences. The search is rapid and is fully customizable, with a choice of FASTA and BED output formats.

**Availability and implementation:**

orfipy is implemented in python and is compatible with python v3.6 and higher. Source code: https://github.com/urmi-21/orfipy. Installation: from the source, or via PyPi (https://pypi.org/project/orfipy) or bioconda (https://anaconda.org/bioconda/orfipy).

**Supplementary information:**

Supplementary data are available at *Bioinformatics* online.

## 1 Introduction

Open reading frames (ORFs) are sequences that have potential to be translated into proteins. They are delineated by start sites, at which translation is initiated by assembly of a ribosome complex, and stop sites, at which translation is terminated and the ribosome complex disassembles (Sieber *et al.*, 2018).

Accurate annotation of the protein coding regions in sequenced genomes remains a challenging task in bioinformatics. For simpler prokaryotic genomes, ORFs correspond to the potential coding sequences (CDS) (Sieber *et al.*, 2018). In eukaryotes, where gene splicing is prevalent, eukaryotic CDS prediction a much more challenging task (Seetharam *et al.*, 2019; Sieber *et al.*, 2018).

Transcriptomic data is critical in addressing this challenge, where presence of an ORF in a mature tranrscript may indicate a potential protein coding gene (Mahmood *et al.*, 2020; Martinez *et al.*, 2020; Seetharam *et al.*, 2019). These data are key to identifying potential orphan genes (Seetharam *et al.*, 2019), young genes unique to a species (Singh and Wurtele, 2020; Tautz and Domazet-Lošo, 2011; Vakirlis *et al.*, 2020); standard *ab initio* gene-prediction models are trained on canonical gene features and do not work well for identifying orphan genes, which are often sparse in canonical gene features (Heames *et al.*, 2020; Ruiz-Orera *et al.*, 2015; Seetharam *et al.*, 2019).

Depending on data (genomic, transcriptomic or metagenomic) and researcher interest, the computational problem of ORF prediction may be stated in multiple ways (Sieber *et al.*, 2018), yet existing tools lack the flexibility to allow users to fine-tune or customize the search for ORF sequences. Here we present orfipy, an efficient tool for extracting ORFs from nucleotide sequences. orfipy provides rapid, flexible searches in multiple output formats to allow easy downstream analysis of ORFs.

## 2 Implementation

orfipy is written in python, with the core ORF search algorithm implemented in cython to achieve faster execution times. orfipy uses the pyfastx library (Du *et al.*, 2020) for efficient parsing of input FASTA/FASTQ file. orfipy can leverage multiple cpu-cores to process FASTA sequences in parallel, based on available memory and cpu cores (Supplementary Data).

### 2.1 Input, flexible search and output

**Fig. 1.**
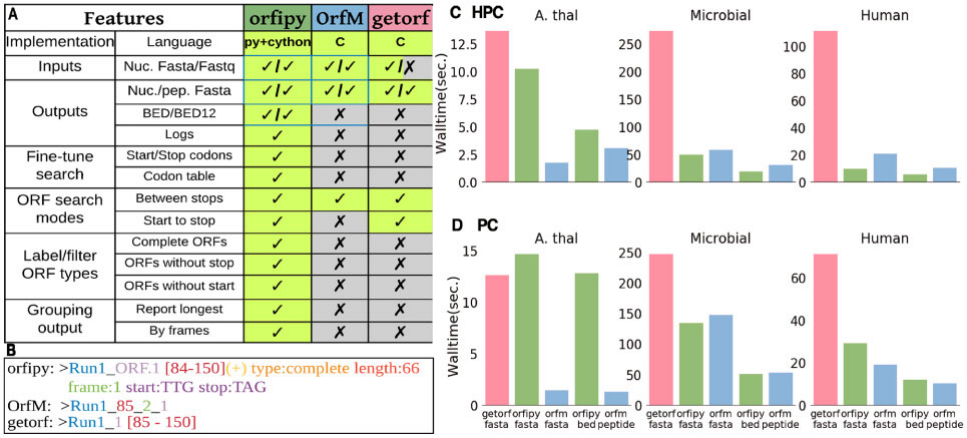
Comparison of orfipy features and performance with getorf and OrfM. We compared attributes of orfipy with two commonly used tools for ORF identification. (**A**) Comparison of orfipy features with getorf and OrfM. orfipy provides a number of options to fine-tune ORF search, this includes labeling the ORF type, reporting only the longest ORF and reporting ORFs by translation frame. To allow reproducible analysis, orfipy logs the commands. (**B**) Example of FASTA headers written to output files by each tool. orfipy output provides information about each ORF that can be readily used in downstream analyses. (**C and D**) Runtimes, using plain FASTA input, on HPC (128 GB RAM; 28 cores) (C) and PC (16 GB RAM; 8 cores) (D) environments (Supplementary Data). Each analysis was run three times, via pyrpipe (Singh *et al.*, 2020), and the mean runtime is reported. orfipy runtimes are comparable to OrfM for the large microbial and human transcriptome data. orfipy is fastest when ORFs are saved to a BED file; OrfM is fastest when ORFs are saved to peptide FASTA. Data sizes: *A.thaliana* genome 120 MB; microbial sequences 1.5 GB; human transcriptome 370 MB. fasta, output ORFs to nucleotide *and* peptide FASTA; bed, output ORFs to BED file; peptide, output ORFs to peptide-only FASTA

orfipy takes nucleotide sequences in a multi-FASTA/FASTQ, plain or gz-compressed, file as input. Users can provide input parameters that include minimum and maximum size of ORFs, list of start and stop codons and/or a user-defined codon table (Supplementary Data). For efficient and flexible downstream analysis (Fig. 1A, B), orfipy provides multiple output types including BED format. BED files reduce disk space use by storing only the coordinates of the ORFs, and are useful in developing more scalable, flexible downstream analysis pipelines. orfipy also adds relevant information about codon use and ORF types, and can group the output by longest ORF contained in each transcript, or can list each reading frame in each transcript.

orfipy enables researchers to fully fine-tune ORF searches using a variety of options (Fig. 1A). For example, users can limit ORF searching to a specific start codon or choose to output ORFs without an inframe start codon. orfipy labels each ORF for users to easily comprehend results (Supplementary Data).

### 2.2 Comparison with existing tools

We compared orfipy with two popular ORF searching tools, getorf (Rice *et al.*, 2000) and OrfM (Woodcroft *et al.*, 2016). What sets orfipy apart is its flexibility and the options to fine-tune ORF searches and output (Fig. 1A, B). Runtimes (Fig. 1C, D) depend on software, environment, input (FASTA input is shown) and output-type. In all scenarios except using a PC to analyze the *A.thaliana* genome, orfipy is much faster than getorf, and comparable to OrfM, with OrfM being faster for FASTQ input (Supplementary Data).

## Supplementary Material

btab090_Supplementary_Data
