# Supplementary material for: orfipy: a fast and flexible tool for extracting ORFs
Source: Bioinformatics. 2021 Feb 8;37(18):3019–20. doi: 10.1093/bioinformatics/btab090 (PMC8479652; doi:10.1093/bioinformatics/btab090)
Supplement: btab090_Supplementary_Data [file btab090_supplementary_data.pdf]

orfipy: a fast and flexible tool for extracting ORFs (Supplementary Materials)

Urminder Singh[1,2,3] **and Eve Syrkin Wurtele**[1,2,3*]

[1]Bioinformatics and Computational Biology Program, Iowa State University, Ames, IA 50011, USA
[2]Center for Metabolic Biology, Iowa State University, Ames, IA 50011, USA
[3]Department of Genetics Development and Cell Biology, Iowa State University, Ames, IA 50011, USA
[*]e-mail: mash@iastate.edu

Getting started

Current stable version of `orfipy` can be installed via PyPI using the following command

```
pip install orfipy
```

Anaconda users can install `orfipy` from the bioconda channel.

```
conda install -c bioconda orfipy
```

Information on installing the development version from the source code is available from https://github.com/urmi-21/orfipy. During submission of this manuscript, `orfipy` version 0.0.3 source code can be accessed from: https://doi.org/10.5281/zenodo.4404795

Usage

`orfipy` requires a fasta/multi-fasta/fastq file, in plain or gzipped format, as input to search ORFs. Other several options can be used to fine tune the ORF search. Basic command to execute `orfipy` looks like:

```
orfipy [<options>] <in.fa>
```

`orfipy` help menu, which describes all the input options, can be printed using the command:

```
orfipy -h
```

Providing start and stop codons and a translation table

With `orfipy`, users can specify one of the 23 codon tables from NCBI (https://www.ncbi.nlm.nih.gov/Taxonomy/Utils/wprintgc.cgi?chapter=cgencodes) to use for translation. If needed, users can also input their own table with the –table parameter. The codon table should be in a .json file to be read by orfipy. An example is provided here: https://raw.githubusercontent.com/urmi-21/orfipy/master/scripts/example_user_table.json

By default, the codon tables specify the start and stop codons to use. Users can easily override these values using *– –start* and *– –stop* parameters when running orfipy. For example:

```
orfipy in.fasta --start TTG,CTG #uses TTG and CTG start codons
or
orfipy in.fasta --start ATG --stop TAA #uses only TAA as stop codon
```

ORF searching modes

`orfipy` has two ORF searching modes by which users can search for ORFs. A user can choose either one.

1. Between Start and Stop: ORFs are defined as the regions between a start and a stop codon. For example, consider the sequence

   AAAATGTTTAAAGGGCCCTAGTTT

   The ORF starting with the start codon "ATG" and ending with the stop codon "TAG" (stop codon not included) is:

   ATG TTT AAA GGG CCC

2. Between Stops (regions free of stop codon): ORFs are defined as regions free of stop codons. For example, for the sequence

   AAAATGTTTAAAGGGCCCTAGTTT

   ORFs reported in frame +1 will now be:

   ```
   AAA ATG TTT AAA GGG CCC
   and
   TTT
   ```

ORF types

`orfipy` labels each ORFs as one of the following:

1. **complete**: This type of ORF has both a start and a stop codon. For example:

   ATG TTT AAA GGG CCC TAG (STOP)

   (the stop codon is included in the ORF for reference)

2. **5-prime partial**: This type of ORF has a stop codon but lacks a start codon. For example:

   TTT AAA GGG CCC TAG (STOP)

   (the stop codon is included in the ORF for reference)

3. **3-prime partial**: This type of ORF has a start codon but lacks a stop codon. For example:

   ATG TTT AAA GGG CCC

By default `orfipy` outputs all **complete** ORFs. Users can use the flags $--partial-5$ and $--partial-3$ to include the **5-prime partial** and **3-prime partial** ORFs.

API to orfipy

We have provided a handy function, "orfipy_core.orfs()" for users to directly use orfipy's ORF search algorithm in python. This section provides a small example.

```
import orfipy_core
seq='ATGCATGACTAGCATCAGCATCAGCAT'
orfipy_core.orfs(seq,minlen=3,maxlen=1000)
```

The above command generates a list of all ORFs found in the sequence passed to orfs() function. The orfs() function can take a number options to fine-tune ORF searches. More details are provided on orfipy's GitHub repository.

Implementation Details

`orfipy` is written in python, with the core ORF search algorithm implemented in cython to achieve faster execution times. `orfipy` takes nucleotide sequences in a multi-fasta or fastq file as input. The files can be plain or in gzipped compressed format. Using the python package pyfastx[1], `orfipy` iterates over the input fasta or fastq file for easy and efficient access to the input sequences.

`orfipy` efficiently searches for the start and stop codon positions in a sequence using the Aho–Corasick string-searching algorithm via the pyahocorasick library (`https://pypi.org/project/pyahocorasick/`).

`orfipy` uses python's multiprocessing library to to process multiple sequences in parallel. The number of sequences to be processed in parallel is automatically estimated by `orfipy` at runtime, as based on available memory and cpu cores. This ensures optimal performance and limits memory usage.

Memory considerations

`orfipy` loads multiple fasta/fastq sequences into memory, and searches for ORFs in parallel. This approach is particularly fast for files containing multiple shorter sequences, such as *de-novo* transcriptome assembly data.

Files with extremely long sequences, such as whole genomes, can require more memory due to the number of ORFs per sequence (which is roughly proportional to sequence length). However, this increased memory use and resultant slower system performance can be avoided. The user has an option to set the $--chunk-size$ option to control memory usage. `orfipy` will then process the input file in smaller chunks of size $--chunk-size$. By default `orfipy` is able to determine an optimal value of $--chunk-size$, based on system's available memory, cpu cores and the input file provided.

Benchmarking details

To compare the runtimes of `orfipy` with getorf[2] and OrfM[3], we used three datasets of different sizes and structures: *A. thaliana* whole genome (TAIR 10), 5,000 microbial genomes and RNA, and Human transcriptome (Ensembl release-101). The $--chunk-size$ and $--procs$ parameters were not supplied to `orfipy` and were automatically estimated by `orfipy` during runtime.

Each tool was run to find ORFs defined as regions free of stop codons, because OrfM supports only this mode. Each tool was run to save ORFs as nucleotide *and* peptide fasta files. OrfM was also run to output ORFs to only peptide fasta file. Additionally, `orfipy` was also run to output ORFs in BED format (the other tools do not provide this option).

For *A. thaliana*, the minimum ORF size was set as 300; for the other two datasets, the minimum ORF size was set to 99. A script was written to execute each of the three tools via pyrpipe[4] to capture the runtimes. Tools were run three times and mean runtime for each tool is reported. Code to download data and run the benchmarks is available at `https://github.com/urmi-21/orfipy/tree/master/scripts`.

Basic commands for executing tools looked like the following:

orfipy

```
orfipy --min <m> --dna <nuc.fa> --pep <pep.fa> --between-stops <in.fa>
and
orfipy --min <m> --bed <out.bed> --between-stops <in.fa>
```

OrfM

```
orfm -m <m> -t <nuc.fa> <in.fa> > <pep.fa>
```

getorf

```
getorf -find 0 -min <m> -outseq <pep.fa> -sequence <in.fa>
getorf -find 2 -min <m> -outseq <nuc.fa> -sequence <in.fa>
```

We compared the ORFs reported by each tool, using https://github.com/urmi-21/orfipy/blob/master/scripts/compare_fasta_files all three tools reported identical ORFs.

System information

We used two different systems for running the benchmark comparisons. The first was a PC running Debian 9.12 (64 bit) with 16 GB RAM and Intel Core i7-3770 CPU. The second, an HPC, was a regular memory node on Pittsburgh Supercomputing Center's Bridges system. Bridges RM node had 128GB RAM and 2 Intel E5-2695 v3 CPUs providing a total of 28 CPU cores (https://www.psc.edu/resources/bridges/system-configuration/).

All the tools were installed via conda. To allow for reproducing the conda environment we have provided the conda environment information as .yml files available from https://github.com/urmi-21/orfipy/tree/master/scripts.

Compatibility with other tools

This section describes how to set `orfipy` parameters to extract the same ORFs as with some existing tools. This will be helpful to users wanting to switch to orfipy from existing tools.

OrfM

OrfM[3] has only one run mode i.e. find ORFs defined as free of stop codons. To use `orfipy` to extract these ORFs, use the flag $--between-stops$. OrfM command:

```
orfm -m <m> -t <nuc.fa> <in.fa> > <pep.fa>
```

`orfipy` command:

```
orfipy --min <m> --dna <nuc.fa> --pep <pep.fa> --between-stops <in.fa>
```

getorf

getorf[2] can find ORFs defined as regions free of stop codons or regions between start and stop codons. User can choose among these options by using the $-find$ parameters in getorf. When $-find$ is set to 0 or 2 (0 for peptide output and 2 for nucleotide), getorf extracts ORFs defined as regions free of stop codons. This is same as OrfM and `orfipy` command will be identical to what described for OrfM i.e. using the $--between-stops$ flag.
When $-find$ is set to 1 or 3 (1 for peptide output and 3 for nucleotide), getorf extracts ORFs defined as regions between start and stop codons. However, getorf only uses ATG as the start codon and also outputs ORFs lacking a stop codon. Thus, to get same ORFs as getorf from `orfipy`, users can specify the start codon to be ATG and to include 3-prime partial ORFs (ORFs lacking a stop codon).
getorf:

```
getorf -find 3 -min $3 -outseq "$2/getorf_3_d" -sequence $1
```

Equivalent `orfipy` command will be:

```
orfipy --min <m> --dna <nuc.fa> --start ATG --partial-3 <in.fa>
```

TransDecoder

The TransDecoder[5] tool can be used for inference of candidate coding sequences. *TransDecoder.LongOrfs* command identifies ORFs in sequences. Then, using an algorithm that predicts well- conserved genes, *Transdecoder.Predict* computes a log-likelihood score for each ORF, and predicts the likely coding regions using the score- and length-based filtering criteria. This filtering out of non-canonical ORFS is not done by orfipy, orfM, or GetOrf. *Transdecoder.Predict* outputs the ORF coordinates in a BED12 format. `orfipy` can out output ORFs in the same format, by choosing out type as BED12 and using *includestop* flag.

```
orfipy --bed12 <orfs.bed> --start ATG --partial-3 --partial-5 --include-stop <in.fa>
```

Note: Since TransDecoder applies a log-likelihood score-based criteria to select those ORFs predicted to encode CDS, the number of ORFs reported will be less than that for `orfipy`, and many ORFs encoding novel CDS may be missed.

Supplementary Figures

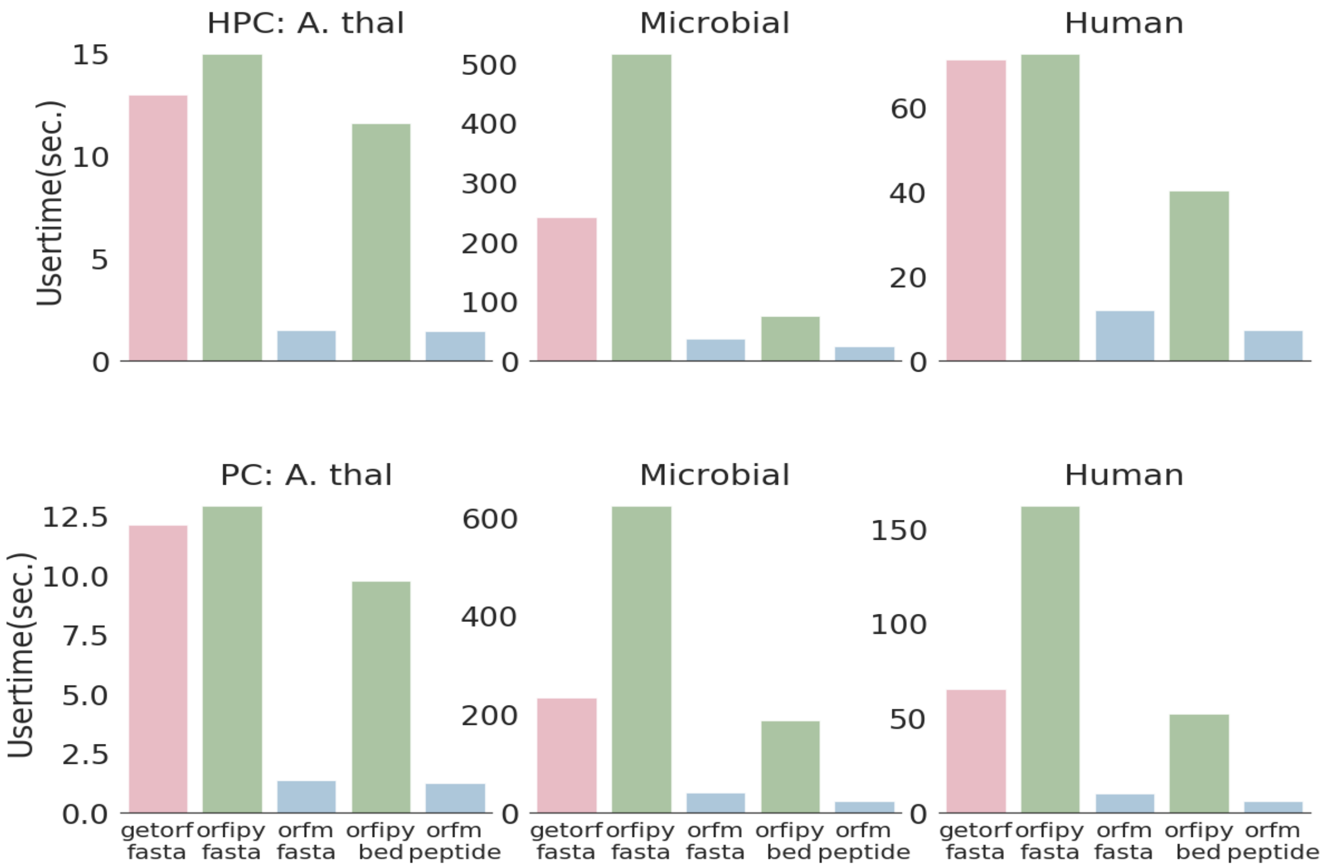

Supplementary Figure 1. Comparison of user CPU times for `orfipy`, `getorf` and `OrfM` on HPC (128 GB RAM; 28 cores) and PC (16 GB RAM; 8 cores). The times were measured using the "time" command. Since `orfipy` takes advantage of multiple CPU cores, total user CPU time is generally higher than the other tools. **Note:** for all data tested, CPU usage of `orfipy` is such that even a standard PC can easily handle it. **Data sizes:** *A. thaliana* genome 120 MB; microbial sequences 1.5 GB; human transcriptome 370 MB. Tools were run to output fasta: output ORFs to nucleotide *and* peptide FASTA; bed: output ORFs to BED file; peptide: output ORFs to peptide-only FASTA.

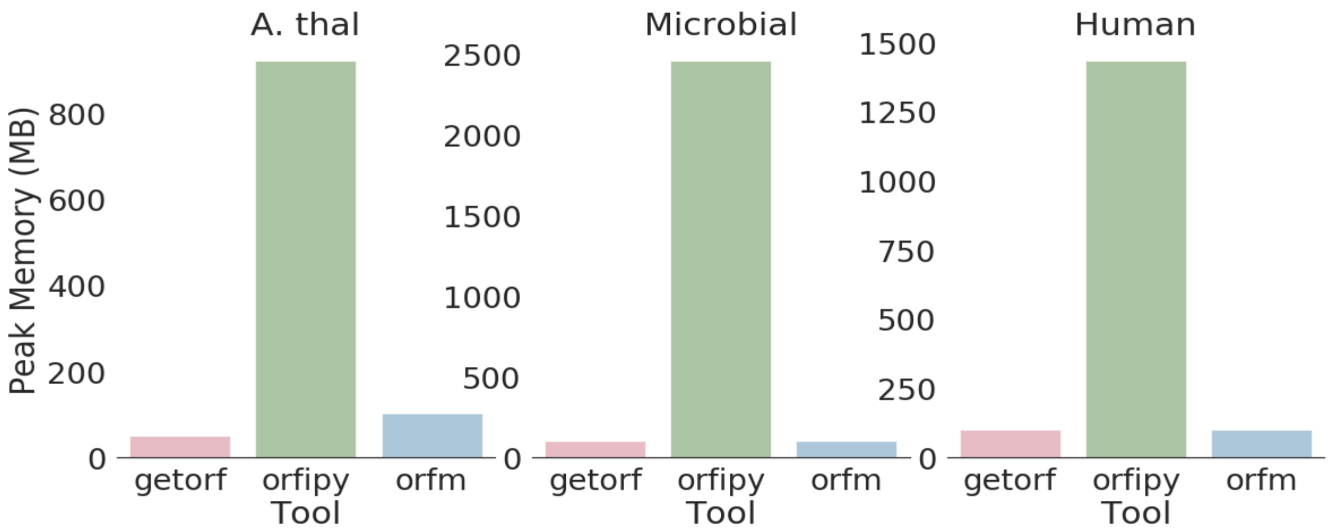

Supplementary Figure 2. Comparison of peak memory usage times for `orfipy`, `getorf` and `OrfM`. The memory usage was monitored using the "System Monitor" program on Debian Linux. `orfipy` loads multiple sequences in memory for parallel processing, and thus has much higher memory usage as compared to the other tools. All tools were run to output ORFs in nucleotide and peptide fasta format. **Note:** for all data tested, peak memory usage of `orfipy` is such that even a standard PC can easily handle it

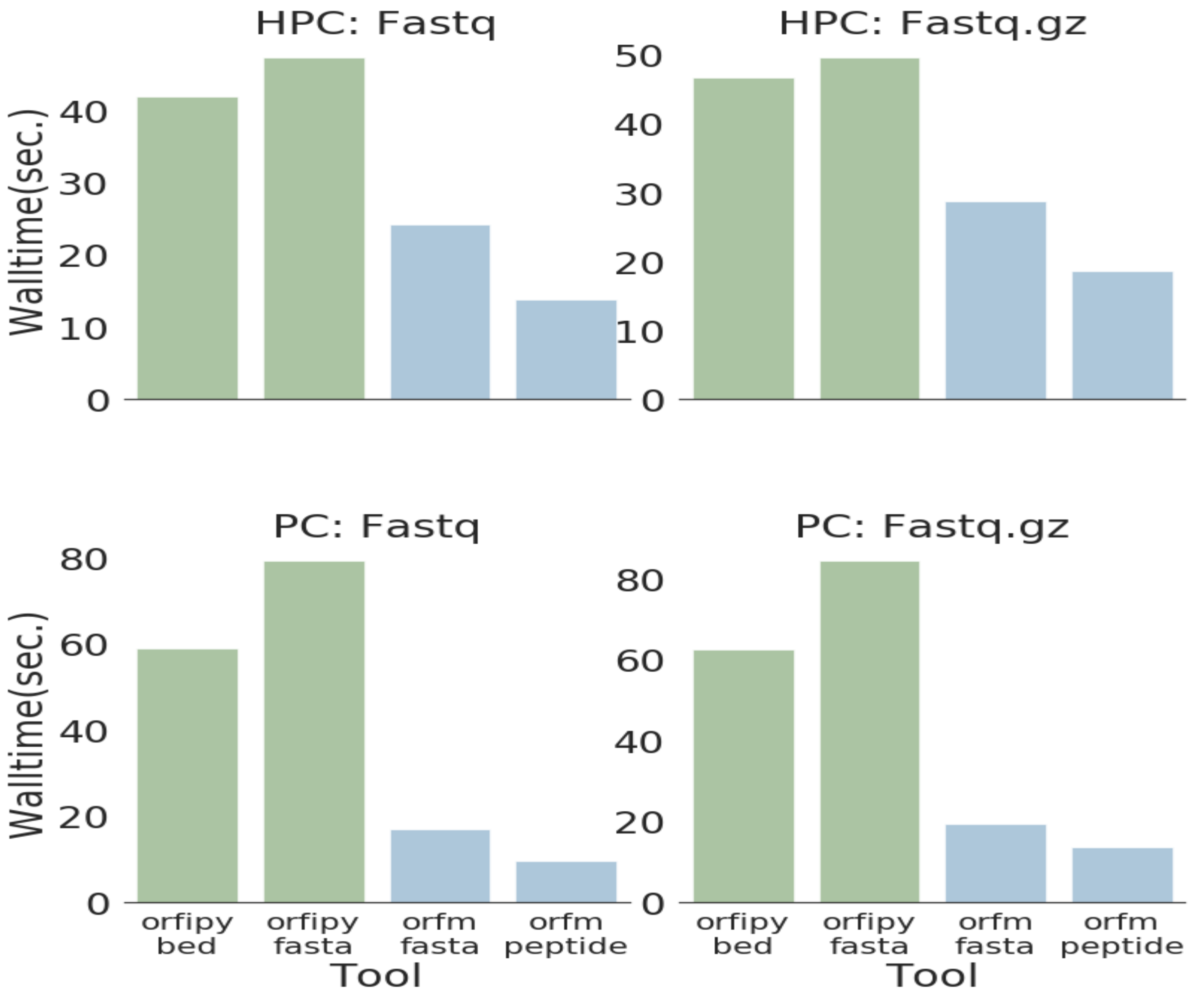

Supplementary Figure 3. Comparison of walltimes times for `orfipy`, and `OrfM` when Fastq or gzipped Fastq file are used for input. The input Fastq was downloaded from NCBI-SRA (SRR976159_1.fastq). `OrfM` performs faster than `orfipy` when Fastq file is provided as an input. `orfipy` and `OrfM` were run to output ORFs in nucleotide and peptide fasta format.

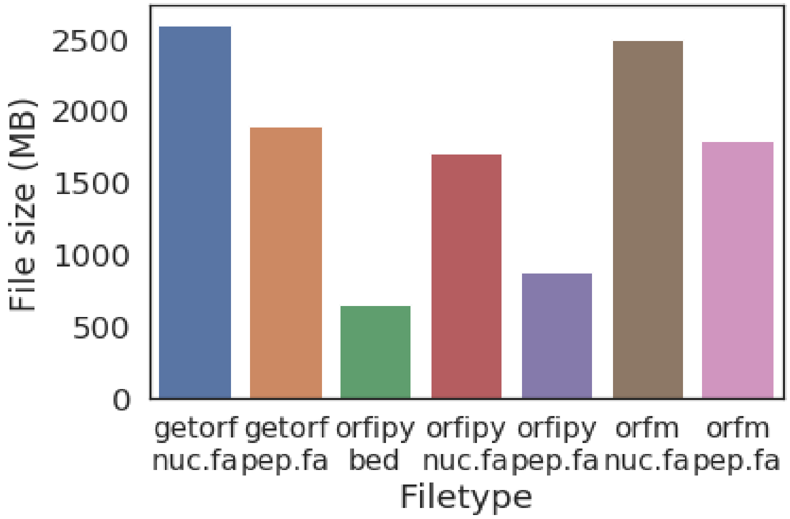

Supplementary Figure 4. Comparison of different output file sizes by `orfipy`, `getorf` and `OrfM`. Each tool was run on the human transcriptome data used in the benchmark comparison. BED file supported by only `orfipy` has the smallest file size. `orfipy` writes smaller Fasta headers hence fasta files generated by `orfipy` in this scenario are much smaller.

References

1. Du, L. *et al.* Pyfastx: a robust python package for fast random access to sequences from plain and gzipped fasta/q files. *Briefings Bioinformatics* (2020).
2. Rice, P., Longden, I. & Bleasby, A. Emboss: the european molecular biology open software suite (2000).
3. Woodcroft, B. J., Boyd, J. A. & Tyson, G. W. OrfM: a fast open reading frame predictor for metagenomic data. *Bioinformatics* **32**, 2702–2703, DOI: 10.1093/bioinformatics/btw241 (2016). https://academic.oup.com/bioinformatics/article-pdf/32/17/2702/17345985/btw241.pdf.
4. Singh, U., Li, J., Seetharam, A. & Wurtele, E. S. pyrpipe: a python package for rna-seq workflows. *bioRxiv* DOI: 10.1101/2020.03.04.925818 (2020). https://www.biorxiv.org/content/early/2020/04/08/2020.03.04.925818.full.pdf.
5. Haas, B. & Papanicolaou, A. Transdecoder (2017).
